# Open notes in psychotherapy: An exploratory mixed methods survey of psychotherapy students in Switzerland

**DOI:** 10.1177/20552076241242772

**Published:** 2024-03-28

**Authors:** Anna Kharko, Sarah Buergler, Annika Bärkås, Maria Hägglund, Jens Gaab, Asbjørn Johansen Fagerlund, Cosima Locher, Charlotte Blease

**Affiliations:** 1 Department of Women's and Children's Health, 8097 Uppsala University, Uppsala, Sweden; 2 Faculty of Health, 6633 University of Plymouth, Plymouth, UK; 3 Division of Clinical Psychology and Psychotherapy, Faculty of Psychology, 27209 University of Basel, Basel, Switzerland; 4 Digital Health Services, Norwegian Centre for E-health Research, Tromsø, Norway; 5 Department of Consultation-Liaison Psychiatry and Psychosomatic Medicine, University Hospital Zurich, University of Zurich, Basel, Switzerland; 6 Department of General Medicine, Beth Israel Deaconess Medical Center, 1811 Harvard Medical School, Uppsala, Sweden

**Keywords:** Open notes, online record access, patient-centered care, psychotherapy, survey, qualitative survey, clinical psychology, electronic health records, healthcare ethics, informed consent, autonomy

## Abstract

**Background:**

In a growing number of countries, patients are offered access to their full online clinical records, including the narrative reports written by clinicians (the latter, referred to as “open notes”). Even in countries with mature patient online record access, access to psychotherapy notes is not mandatory. To date, no research has explored the views of psychotherapy trainees about open notes.

**Objective:**

This study aimed to explore the opinions of psychotherapy trainees in Switzerland about patients’ access to psychotherapists’ free-text summaries.

**Methods:**

We administered a web-based mixed methods survey to 201 psychotherapy trainees to explore their familiarity with and opinions about the impact on patients and psychotherapy practice of offering patients online access to their psychotherapy notes. Descriptive statistics were used to analyze the 42-item survey, and qualitative descriptive analysis was employed to examine written responses to four open-ended questions.

**Results:**

Seventy-two (35.8%) trainees completed the survey. Quantitative results revealed mixed views about open notes. 75% agreed that, in general open notes were a good idea, and 94.1% agreed that education about open notes should be part of psychotherapy training. When considering impact on patients and psychotherapy, four themes emerged: (a) negative impact on therapy; (b) positive impact on therapy; (c) impact on patients; and (d) documentation. Students identified concerns related to increase in workload, harm to the psychotherapeutic relationship, and compromised quality of records. They also identified many potential benefits including better patient communication and informed consent processes. In describing impact on different therapy types, students believed that open notes might have differential impact depending on the psychotherapy approaches.

**Conclusions:**

Sharing psychotherapy notes is not routine but is likely to expand. This mixed methods study provides timely insights into the views of psychotherapy trainees regarding the impact of open notes on patient care and psychotherapy practice.

## Introduction

In the past decade, health institutions in around 30 countries have begun to provide patients with online access to their medical records via secure portals and apps.^1^ Access includes test results, lists of medications, and even the narrative reports written by clinicians (the latter, often referred to as “open notes”). Open notes are associated with a range of benefits for patients, including an enhanced engagement and recall about their care plans.^2,3^ In some countries, such as Sweden and the US, the practice is advanced with most patients offered full, prompt online access to most of their clinical records.^4,5^ In Switzerland, organizational networks of health professionals and their institutions (e.g. hospitals, nursing homes, birth houses, doctors’ practices, pharmacies, Spitex services, and rehabilitation clinics or therapists) have also begun to provide online record access (ORA) (https://www.patientendossier.ch). They are usually called electronic patient file (“elektronisches Patientendossier”; EPD) in Switzerland.

Despite advances that are associated with open notes, many countries including those with digitalized health records have not implemented patient access. In the case of Canada and Germany, for example, open notes are available to some patients; however, they are not offered universally.^1^ In China, some hospitals provide inpatients with ORA.^6^ In South Korea, through the MyHealthWay app by 2024, it is expected that all health records data, including open notes, will be integrated into the app.^7^ Elsewhere in the EU, in Bulgaria, online patient access to the health record was recently launched at the end of 2022 with prospective access to open notes.^8^ In Switzerland, a country with 8.927 million inhabitants, fewer than 20,000 EPD dossiers have been opened throughout the country so far.^9^

Psychotherapy is a collective term for varying methods of providing mental healthcare, via so-called talk therapies. It can be delivered in inpatient, out-patient, remote, and ambulatory settings. In this study, psychotherapy notes refer to the qualitative clinical documentation from a psychotherapy session. The sharing of psychotherapy notes remains controversial. For example, in the US from April 2021, the twenty-first Century Cures Act mandated that providers offer patients access to their online clinical records, without charge; however, psychotherapy notes are exempt from this ruling.^10^ In Sweden, the Swedish National Regulatory Framework states that patients must be able to access their health information, including their notes regardless of whether they were produced in mental healthcare or general practice.^11,12^ Some exceptions apply and are related to the safety of the patient. In practice, due to the decentralization of healthcare in Sweden, each region decides how to interpret the regulations. In 2021, five of the 21 regions did not routinely provide access to psychiatric notes.^11^ In Norway, the national provider of ORA makes no distinction between psychotherapy notes and other documentation in the medical record.^13^ Consequently, in regions that have implemented ORA, patients have access to both their structured documentation such as referrals and discharge notes, and their free form narrative documentation (“open notes”). Although not mandated by law, most public healthcare institutions provide this access, while private healthcare providers usually do not. In Switzerland, inpatient psychiatric clinics are obliged to provide EPDs, including open notes^14^; in contrast, for ambulatory psychiatric services and psychotherapists participation is still voluntary.^15^

The controversies around sharing psychotherapy notes are understandable: offering patients access to open notes can be reformulated as a dilemma balancing patient autonomy with the possible risks of harm from reading the in-depth documentation written by therapists.^16,17^ Scarce attention has been given to the opinions and views of mental health clinicians, especially of psychotherapists, with respect to opening notes to patients,^18–22^ and limited research has been conducted on the experiences of patients reading their psychotherapy notes.^23–26^ More generally, where studies exist, the findings are mixed. Many mental health clinicians—especially those working in psychiatric and psychotherapy settings—remain concerned that patients may become anxious, confused or offended by what they read, and that making notes accessible to patients could exacerbate work burdens.^18,27–29^ For example, in the US, in a survey conducted with the Department of Veterans Affairs (VA)—the nationwide health system that provides all enrolled veterans with access to their mental health notes—around half of surveyed mental health clinicians reported they would be “pleased” if open notes were discontinued.^29^ In Sweden, nearly two in three clinical psychologists and a third of psychiatrists reported being less candid in their clinical notes as a result of implementation of the practice.^28^ There exists also the concern about parallel and more complete records that are collected without the patient's knowledge (i.e. called shadow dossier). In Norway, healthcare personnel in an out-patient mental health setting reported that their documentation practices had changed over time, but they were not sure whether to attribute this to patients having access to their notes.^30^ In another study comparing mental and somatic healthcare (the latter referring to physical healthcare needs), a higher proportion of healthcare personnel in mental healthcare than in somatic healthcare reported having changed their writing after the implementation of ORA.^31^ A study from Norway found that up to a third of healthcare professionals in psychiatry underreport information in the patient record, compared to a fifth of their colleagues in somatic care, and almost 1 in 10 of psychiatry healthcare professionals kept a shadow record.^32^

Preliminary research suggests that open notes may benefit patients in mental health settings. For example, in the US, a small pilot study conducted at a psychiatric out-patient clinic found that, after 20 months, most patients reported an increased understanding about their mental health, and better awareness about the potential side effects of medications.^33^ Other larger surveys in the US support the finding that patients with mental health diagnoses report better understanding their medications, and doing a better job taking prescribed medications as a result of open notes.^34^

While mental health care in general has received some attention, research specifically devoted to isolating experiences of open notes in psychotherapy settings is very limited.^22,25^ In the US, a pilot qualitative study conducted at one academic center shows mixed findings demonstrating that surveyed patients felt more in control of their care, and access was extremely important for trusting their provider, remembering what they were working on in therapy, and feeling engaged; however, some patients perceived notes as inaccurate, disrespectful, or judgmental, and strain was more likely if patients reported surprises in the notes, or incongruencies between what was communicated face-to-face and what was documented.^25^ A pilot study of therapists’ experiences at the same center reported participants who agreed to share their notes were generally positive about the innovation.^22^ Notably, however, both studies were limited by small sample sizes and excluded patients with serious mental illnesses, or those therapists with serious misgivings about open notes.^23^ Further limiting the generalizations of these responses, the studies may have been biased in favor of more engaged therapists and patients, perhaps leading to responder biases with more favorable reporting about the practice. Beyond the limited explorations of clinicians’ and patients’ views into sharing open notes, there are scarcely any published investigations into clinical students’ opinions about the practice. Considering that patients’ access to their own health data is unlikely to abate including in mental healthcare, in this study we aimed to explore views about open notes among psychotherapy trainees. This makes the study original in its primacy. We chose to administer the survey in Switzerland, a country where there is a strong tradition of psychotherapy in mental healthcare but where open notes are still only nascent. We identified three research questions:

1. Are psychotherapy students in Switzerland familiar with the concept of open notes?
2. What are the opinions of psychotherapy trainees on the potential impact of open notes on patients?
3. What are the opinions of psychotherapy students on the potential impact of open notes on psychotherapists and the practice of psychotherapy?

## Methods

### Setting and participants

The survey was conducted as a single-centered study at the University of Basel, Switzerland between October 2020 and November 2021. Target participants were (a) students of several psychotherapy training programs at the University of Basel, i.e. Master of Advanced Studies in Person-centered Psychotherapy, Certificate of Advanced Studies in Animal Assisted Therapy, Certificate of Advanced Studies in Motivational Interviewing, Master of Advanced Studies in process-based Psychotherapy (note that psychotherapy students in Switzerland also work as practicing psychotherapists); (b) master students enrolled in the colloquium Clinical Psychology and Psychotherapy at the University of Basel; and (c) practicing psychotherapists with completed training, currently enrolled as students at the Center of Psychotherapy, an ambulant psychotherapy clinic for adults at the University of Basel. In the psychotherapy training programs, the modules cover basic and advanced therapeutic knowledge. Among others, main topics are personality and disorder model; therapy theory and the central importance of the therapeutic relationship; basic therapeutic skills; change processes in psychotherapy; findings from psychotherapy research; and discussion of professional ethics. Psychotherapy training programs have a mean duration of 4 years and encompass regular supervision. Master students who are enrolled in the colloquium are trained in the scientific background of psychotherapy. Those students practicing psychotherapy were mainly employed on an hourly basis in a center for psychotherapy and were also under regular supervision. All potential participants were emailed by the study team with a link to the online survey. Apart from being enrolled as students in the target programs, there were no other inclusion or exclusion criteria for participation.

### Survey

**Figure 1.**
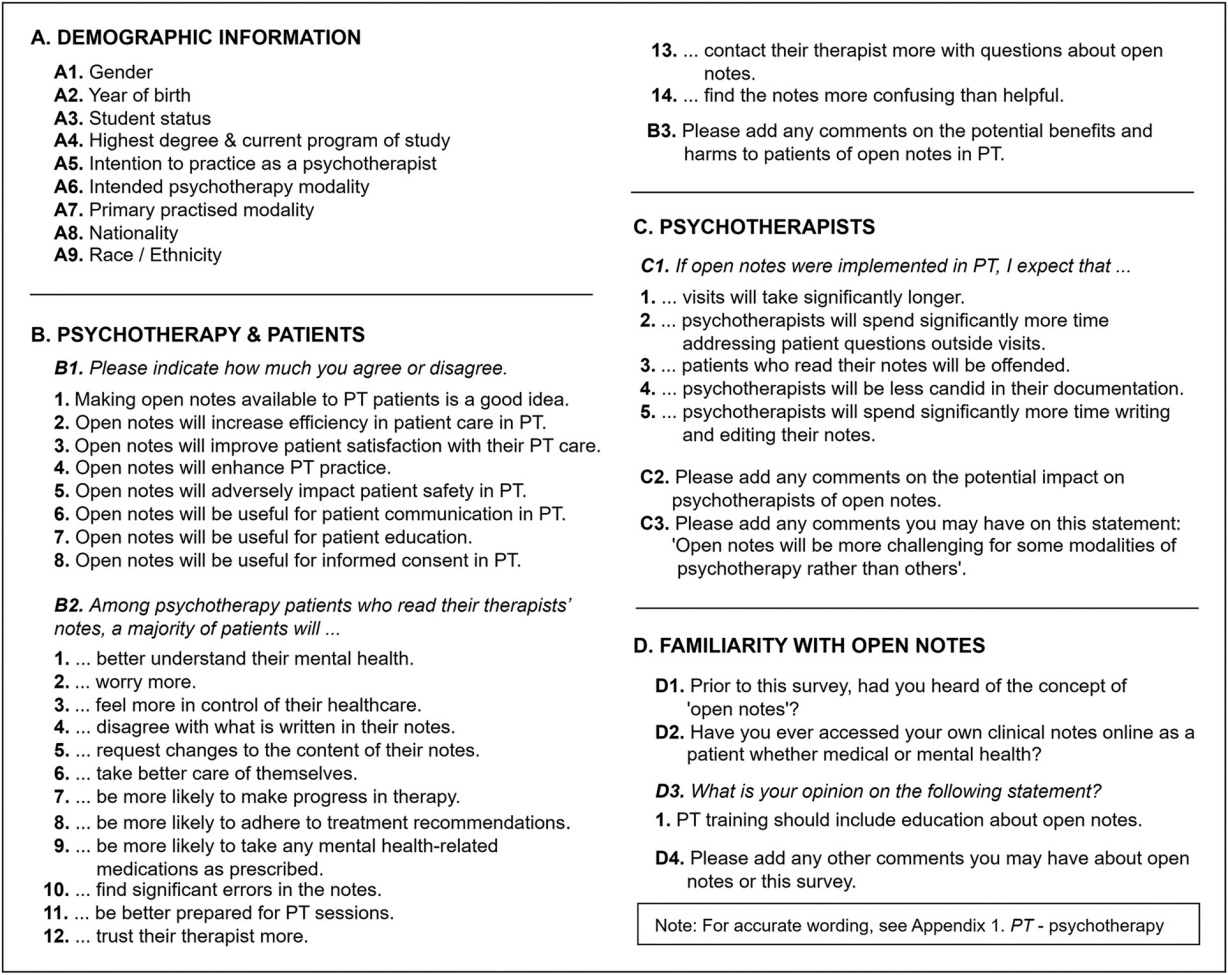
Survey structure. *Note*. PT – Psychotherapy.

The study aims were investigated via an online survey comprised of four sections: One section about demographic information, titled (a) “*Demographic information*,” as well as three sections focusing on open notes in different areas: (b) “*Psychotherapy & Patients*,” (c) “*Psychotherapists*,” and (d) “*Familiarity with open notes”* (see Figure 1). The survey included questions adapted from recent publications on the views of clinicians on open notes in mental healthcare,^27–29^ as well as novel items, specifically tailored to the field of psychotherapy (see Supplementary Material 1).

The survey encompassed 42 items that included 38 closed-ended questions asking for a single- or multiple-choice selection, and four optional open-ended questions, asking for a brief free-text comment. The survey was conducted in Limesurvey (limesurvey.org). The survey was administered in English, but both English and German free-text responses were accepted.

### Data analyses

To answer the first research question, “*Are psychotherapy trainees familiar with the concept of open notes?”*, we analyzed the items in Section D on familiarity and previous experience with open notes. To answer the second research question, “*What potential impact do trainees foresee open notes to have on psychotherapy patients?”*, we analyzed the close-ended items from Section B and conducted qualitative analysis of the free-text comment left in response to Item B3. For the last research question, “*What potential impact do they foresee open notes to have on psychotherapists?”*, we analyzed the closed-ended items in Section C and conducted a qualitative analysis of the free-text comment left in response to Item C2 and C3. Items B3 and C2 were combined for coding and further analysis due to a large overlap in responses.

Quantitative data was analyzed through descriptive statistics, which included averages, standard deviation, absolute values and percentages. The number and frequencies of responses on survey were prepared in Excel (v 16.61) and descriptive statistics and analysis were carried out using RStudio (v 1.2.5003). For qualitative analysis, all comments were included. Any comments that were in German were translated into English by Berfin Bakis and SB. Then, an inductive, thematic, data-driven approach was employed to analyze the comments. SB and AK were main coders who applied the initial codes. AB reviewed the codes independently and created the initial categories. The final categories and themes were adjusted by AK, AB, and SB. The impact of AK, SB, and AB's preunderstanding and prior experiences on the analytical process were reflected upon (see Supplementary Material 2). Initial coding was conducted using QCAmap (https://www.qcamap.org) and the frequency statistics for the final categories and themes were calculated in Excel (v 16.61). To maintain participant anonymity when providing direct quotes, participants were assigned a random individual numerical identifier. The qualitative data was reported following the Standards for Reporting Qualitative Research guideline (Supplementary Material 3).^35^

### Ethical considerations

The study received ethical approval prior to data collection from the Ethics Committee of the Faculty of Psychology, University of Basel, Switzerland (#014-20-3). Informed consent was attained online at the start of the survey. Participants were informed that there is no obligation to participate in the study and that they could choose not to partake in the study without any penalty.

## Results

### Respondents

Out of 201 contacted students, 72 (35.8%) completed the survey. Respondents were aged between 21 years and 60 years with a mean age of 29.2 years (see Table 1).

**Table 1.** Respondents’ characteristics.

	Statistic ^a^
Demographic characteristics	
Age in years, *m* (*SD*)	29.2 (± 6.9)
Gender, *n* (*%*)	
Female	62 (86.1%)
Male	9 (12.5%)
Prefer not to answer	1 (1.4%)
Nationality, *n* (*%*)	
Swiss	49 (68.1%)
Swiss and another nationality ^b^	7 (9.7%)
German	6 (8.3%)
Not reported	10 (13.9%)
Race, *n* (*%*)	
White	62 (86.1%)
Black	1 (1.4%)
Other	2 (2.8%)
Not reported	7 (9.7%)
Highest attained degree, *n* (*%)*	
Bachelors	27 (37.5%)
Masters	43 (59.7%)
Diploma	1 (1.4%)
PhD	1 (1.4%)
Specialty	
Psychology or psychotherapy trainee, *n* (*%)*	57 (79.2%)
Intend to practice as psychotherapist, *n* (*%)*	
Yes	46 (80.7%)
No	1 (1.8%)
Unsure	10 (17.5%)
Of those who intend to practice, intended therapy was …, *n* (*%)* ^c^	
Person-centered therapy	24 (52.1%)
Cognitive behavioral therapy	16 (34.8%)
Integrative therapy	10 (21.7%)
Systemic therapy	10 (21.7%)
Process-oriented therapy	6 (13.0%)
Psychodynamic therapy	6 (13.0%)
Eclectic therapy	2 (4.4%)
Acceptance and commitment therapy	1 (2.2%)
Unclear	1 (2.2%)
Practicing psychotherapists, *n* (*%)*	15 (20.8%)
Practiced therapy, *n* (*%)* ^c^	
Person-centered therapy	11 (73.3%)
Cognitive behavioral therapy	4 (26.6%)
Systemic	3 (20.0%)
Integrative therapy	2 (13.3%)
Psychodynamic therapy	1 (6.6%)
Familiarity and experience with open notes	
Familiar with open notes, *n* (*%)*	28 (41.2%)
Had patient experience with own open notes, *n* (*%)*	8 (11.8%)

^a^ Percentages were calculated excluding missing data.

^b^ Other reported nationalities were Turkish, German, Italian, and Portuguese. To avoid participant identification, reporting of statistics for each dual nationality is omitted.

^c^ Multiple-choice question, for which participants could select more than one answer option. The total number of answers exceeds the number of respondents.

SD: standard deviation.

### Quantitative analyses

**Figure 2.**
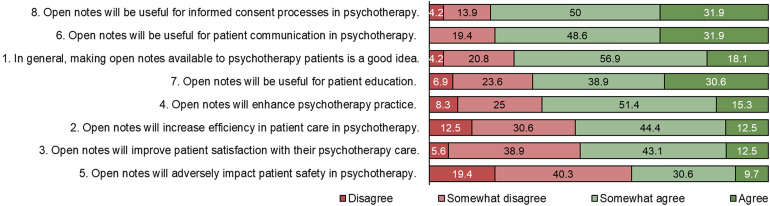
Distribution of responses to statements about the potential impact of open notes on psychotherapy. Ordered from highest agreement (combined answers “*somewhat agree”* and “*agree”*) to lowest. Note: Item 5 is worded in a negative direction which may have affected its rating.

*Impact on psychotherapy.* In general, most students tended to endorse a positive outlook on the impact of open notes on psychotherapy (see Figure 2).

**Figure 3.**
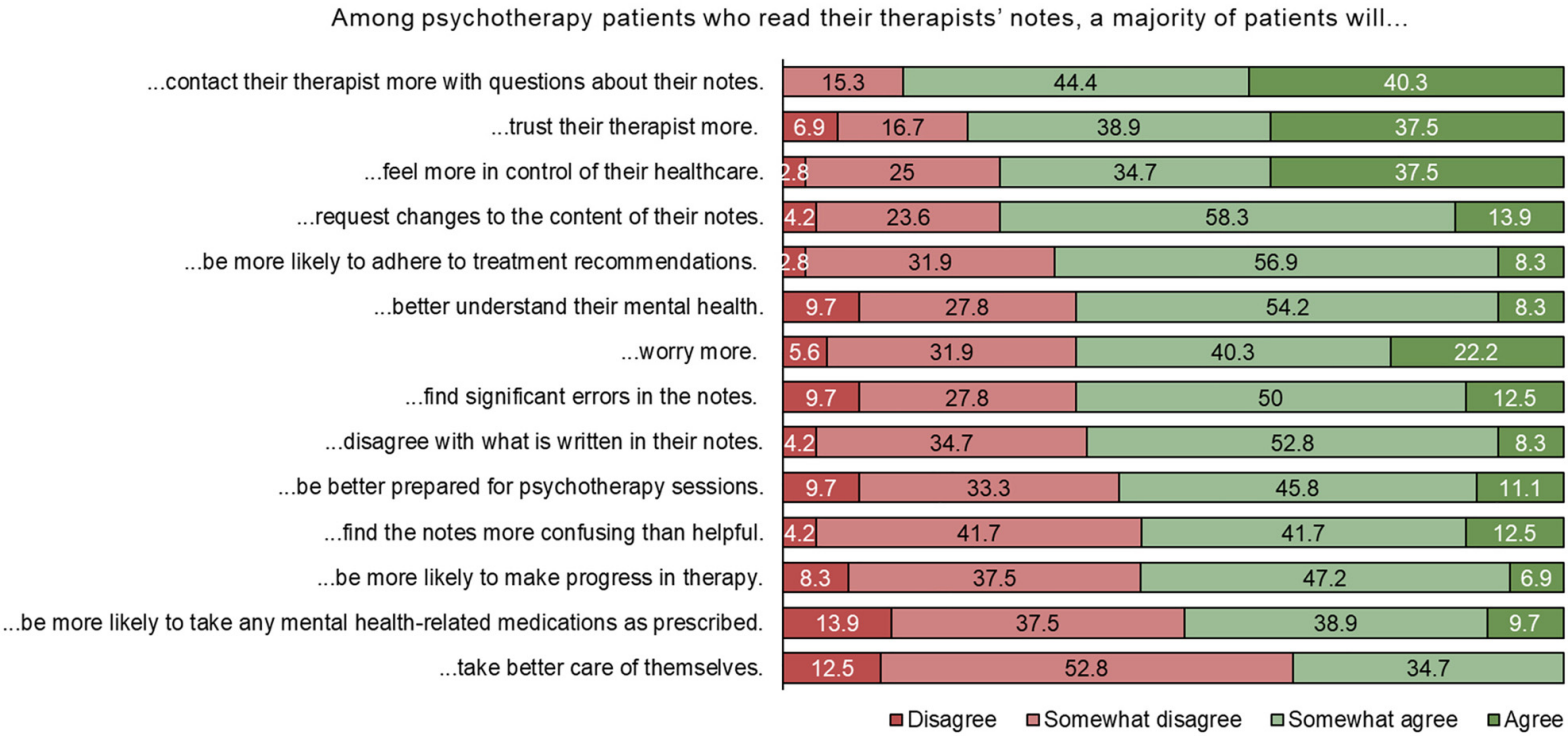
Distribution of responses to statements about the potential impact of open notes on psychotherapy patients. Ordered from highest agreement (combined answers “*somewhat agree”* and “*agree”*) to lowest.

*Impact on psychotherapy patients.* Participants predicted a diverse range of effects of open notes on patients (see Figure 3).

**Figure 4.**
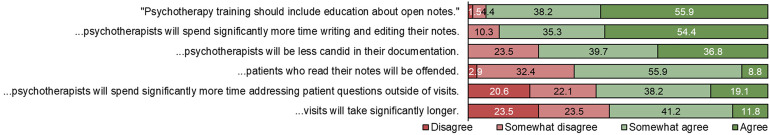
Distribution of responses to statements about the potential impact of open notes on psychotherapists. Ordered from highest agreement (combined answers “*somewhat agree”* and “*agree”*) to lowest.

*Impact on psychotherapists.* Participants forecast divergent, and often negative effects, on the impact of open notes on psychotherapists’ work and documentation practices (see Figure 4). Almost all respondents somewhat agreed or agreed that education about open notes should be part of psychotherapy training (94.1%).

### Qualitative analyses

Respondents left a total of 199 comments. As a result of the qualitative analysis for Items B3, and C2, 242 passages were coded, which gave rise to 23 categories and four major themes (see Table 2). The biggest theme was *Negative impact on therapy* (32%), comprising a variety of predictions about the predicted adverse effects of open notes on therapists’ work as well as the therapeutic process and the patient-therapist alliance. In this theme, over a third of comments predicted an increase in workload, both because of more time spent on documentation as well as on anticipated impacts on therapy sessions (e.g. “*Therapy sessions may take longer”* [Participant #30, female, 29]). Trainees also expected that therapists would feel constrained as they would have “*less freedom in writing down thoughts”* [Participant #17, female, 29], and “*less intuition”* [Participant #38, female, 29]. Some comments suggested open notes could hinder the therapy process either by diverting attention from the patient, harming the course of therapy or the therapeutic alliance itself, for example:

*“Even though therapists are trained to establish and maintain a trustful and honest relationship with their clients, I think the point about honest notes and spending more time to edit notes so that the patient doesn't get offended might disturb this relationship” [Participant #45, male, 24].*

**Table 2.** Themes and categories from qualitative analysis of comments about benefits and harms of open notes to patients and impact on psychotherapists.

Theme & category	*n* (*%*)	Example
Negative impact on therapy	74 (31%)	
Increase workload	26 (35.1%)	*“Spend more time on notes.”*
Therapists will feel pressured/constrained	15 (20.3%)	*“It might put a lot of pressure on therapists to do things right and they might feel policed.”*
Less attention on patient	12 (16.2%)	*“… focus on the notes, less on the actual therapy.”*
Harm the therapy process	12 (16.2%)	*“The therapist's notes could disturb the patient's own developmental process.”*
Harm the therapeutic relationship	9 (12.2%)	*“Could potentially cause conflict.”*
Positive impact on therapy	56 (23%)	
Benefit the therapy process	22 (39.3%)	*“Consent about therapy goals between patient and therapist will be fostered.”*
Increased transparency	13 (23.2%)	*“It shows willingness to transparency on both sides”*
Increased trust	10 (17.9%)	*“Patients would trust their therapist more.”*
Benefit the therapeutic relationship	8 (14.3%)	*“… less hierarchy patient vs. professional.”*
Reduced workload	3 (5.3%)	*“report writing and other administrative tasks could be reduced as it could be co-created during therapy sessions with the patient.”*
Impact on patients	61 (25%)	
Increased anxiety or confusion	19 (31.1%)	*“Patient confusion or anxiety because of technical terms or things they can’t quite evaluate.”*
Impact depends on patient or disorder	17 (27.9%)	*“Whether notes are useful or not depends on patient variables”*
Feeling misunderstood, judged or offended	9 (14.8%)	*“[Patients] maybe misinterpret what is written, might not understand specific words”*
Patient empowerment and autonomy	8 (13.1%)	*“I think it could really help patients’ growth.”*
Feelings of upset or agitation	6 (9.8%)	*“… fear of hurting / upsetting.”*
Increased understanding	2 (3.3%)	*“The notes could help them understand some behaviors or thoughts better.”*
Documentation	51 (21%)	
Changes in note taking	34 (66.7%)	*“I would use different words if the patient can read them at home.”*
Implementation of open notes	8 (15.7%)	*“It makes sense to study what parts of the notes and under which circumstances make sense to share and what the effects are.”*
Need for shadow notes	5 (9.8%)	*“The concept of open notes might be good, but they would have to be specially written for patients and not the clinical notes therapists write for themselves.”*
No change in note taking	4 (7.8%)	*“… there actually is a right to see records- so you should always write your notes as if the patient would read it anyway.”*

*Note*: Percentages for themes were calculated based on the total number of coded passages and percentages for categories were calculated based on the number of coded passages in that theme.

In contrast, a quarter of all coded passages pointed to *Positive impact on therapy* (23%). In this theme, most comments identified a variety of potential benefits of open notes to the therapy process (39.3%), e.g. “*it could make the process more structured”* [Participant #71, female, 25] or “*it may be helpful to reflect on the progress made so far”* [Participant #25, female, 24]. Specific predicted benefits such as an increase in trust and transparency were common. However, fewer comments expected improvements to the therapeutic relationship (14.3%) and only a few hypothesized a reduction in workload (5.3%).

Impact on the patient themselves was discussed in a quarter of all coded passages (*Impact on patients*, 25%). This theme contained more negative predictions than neutral or positive ones. For example, almost a third of passages anticipated an increase in anxiety or confusion among patients (31.3%). This was often linked to anticipations that patients would be unable to understand the written notes due to technical jargon (e.g. “*… patients will worry about the content, because the professional language is something else than how we talk to clients”* [Participant #36, female, 30]*)*. Participants also predicted that patients would feel more misunderstood or offended after reading their notes (e.g. “*would be more often confronted with offended patients or offended relatives of the patient”* [Participant #41, female, 24]) or become upset, perceiving discrimination (e.g. “*patient could feel stigmatized”* [Participant #41, female, 24]). On the other hand, some trainees (27.9%) suggested that the effects of open notes could be more nuanced, depending on the patients or their diagnosis. Beliefs ranged from the cautious (e.g. “*Depending on the disorder, patients may feel criticized or not equal (to the therapist)”* [Participant #59, female, 40]) to the optimistic (e.g. “*If a patient/client is cognitively functional and wants to get treatment, then I think sharing notes could be hugely beneficial”* [Participant #63, female, 34]). A minority of comments referred to positive changes such as patient empowerment and autonomy (e.g. “*I can see the potential for empowering patients in this practice”* [Participant #64, female, 28]); only two participants explicitly mentioned improved patient understanding because of open notes.

A final emergent theme, *Documentation*, contained opinions about the practice of the documentation in the era of open notes (21%). Here, the largest category (which was also the largest overall) comprised a wide variety of comments that described how note taking practices could change after opening them to the patient (66.7%). Some participants predicted that the notes might have to contain “*more careful entries”* [Participant #68, female, 33] which some linked to “*possible loss of first impressions”* [Participant #27, female, 24], and the attendant concern that therapists would have to “*[pay] closer attention to wording so that there are no misunderstandings”* [Participant #21, female, 33]*.* Two comments explained that notes would have to be simplified (e.g. “*Technical terms should be deleted from the notes, as explaining them to the patients would take a lot of time”* [Participant #57, female, 29]) and some forecast a loss or omission in information altogether, e.g. “*I know, that I would skip some kind of information”* [Participant #14, female, 27]. Four comments stated outright that notes might contain incorrect information, e.g. “*psychotherapists might write fake notes, only to avoid long discussions”* [Participant #44, female, 21]. In the remaining categories of this theme, trainees raised questions about how and when open notes should be implemented, as well as the need for keep shadow notes if open notes were implemented (e.g. “*it could be that therapists would start to have “shadow notes” on the side”* [Participant #63, female, 34]). A minority advocated that “*notes should always be written in a way that patients might read it.”* [Participant #40, male, 26]

*Impact on psychotherapy types.* As a result of the qualitative analysis for Item C3, 48 passages were coded, which were analyzed into three major themes with nine categories (see Table 3). The largest theme, *Therapy factors*, comprised of opinions about whether the use of open notes depended on the type of psychotherapy. Most trainees either provided examples of therapies where open notes would be more difficult to apply (e.g. “*More problematic in psychoanalysis”* [Participant 12, female, 28]) or pointed to differences between therapies (e.g. “*certain … therapy approaches require different amounts of translation work for patients”* [Participant #12, female, 28]). In contradiction, a few commented that open notes implementation would not be different across types of therapies (e.g. “*I already write my notes in a way that patients could/ would be allowed to read them any time.”* [Participant #68, female, 33]) or that it could be easier (e.g. “*I expect less difficulties in CBT.”* [Participant #19, female, 31]). Only three comments suggested that the therapy setting is also a factor. For instance, whether it's during an inpatient care (e.g. “*In in-patient settings, the question arises who takes notes and if the patient can see all the notes from all the therapists and health care workers. I think this probably works best for out-patient settings”* [Participant 63, female, 34]) or in legally prescribed therapy (e.g. “*In a setting where therapy is a legal requirement, it could be very difficult for the patient to understand/ have insight of the notes.”* [Participant 42, female, 28]).

**Table 3.** Themes and categories from qualitative analysis of comments about impact of open notes on types of psychotherapy.

Theme & category	*n* (*%*)	Example
Therapy factors	31 (64.5%)	
Different across therapies	9 (29.0%)	*“I don't see open notes working the same way in forensic psychology or traffic psychology or school psychology.”*
More difficult for some therapies	9 (29.0%)	*“Maybe for modalities using more technical terms open notes are more challenging.”*
No difference across therapies	6 (19.4%)	*“I believe the challenge does not depend on different modalities of psychotherapy.”*
Easier for some therapies	4 (12.9%)	*“Behavioral therapy is designed for transparency anyway.”*
Therapy setting	3 (9.7%)	*“I think in acute inpatient treatments it might be rather difficult, as it is often more about stabilization than psychotherapy per se.”*
Patient factors	15 (31.3%)	
Patient capabilities & needs	5 (33.3%)	*“more challenging [when] the actual therapy issue is a problem that the client does not accept yet or is not aware of it”*
Children & adolescents as patients	5 (33.3%)	*“Therapy notes might not be useful for children but maybe for older teens.”*
More difficult for some diagnoses	5 (33.3%)	*“… especially the ones that interfere with information processing.”*
Therapist factors	2 (4.2%)	
Therapist attitude	2 (100%)	*“I'd say it is not primarily about the type of psychotherapy, but the attitude of the psychotherapist.”*

*Note*: Percentages for themes were calculated based on the total number of coded passages and percentages for categories were calculated based on the number of coded passages in that theme.

The second theme, *Patient factors,* comprised of comments about implementation challenges related to the patients themselves. Some trainees suggested that patient capabilities should be considered when using open notes. These included: the patient's cognitive capacity (e.g. “*Cognitive capacities certainly play a big role here”* [Participant 33, male, 25]), patient's ability to understand their diagnosis (e.g. “*challenging for patients who don't think they could have a mental health problem”* [Participant #41, female, 24]), and patient's motivation (e.g. “*patients that lack the motivation to attend psychotherapy (e.g. offenders)”* [Participant 64, female, 28]). Trainees also considered the additional challenges if the patient is underage: “*difficult in child and adolescent psychotherapy as parents might sometimes disagree with therapist and the child might not want the parents to read the notes”* [Participant 64, female, 28]). The rest of the comments on this theme focused on specific diagnoses where open notes use is more difficult, e.g.: “*I work in acute psychiatry. I simply cannot imagine letting patients read how they were acting during their acute psychosis. Some of the patients would be broken knowing what they did (since they cannot recall).”* [Participant #14, female, 27].

The last theme, *Therapist factors*, contained only one category with two comments which pointed to the therapist's attitude as a key factor in the implementation of open notes; “*I'd say it is not primarily about the type of psychotherapy, but the attitude of the psychotherapist”* [Participant 37, female, 24].

## Discussion

### Main findings

In general, this mixed-methods exploratory study of psychotherapy trainees’ views of open notes revealed a varied picture. Participants anticipated that open notes could have negative effects on patients and on the practice of therapy. For example, more than eight in 10 participants somewhat agreed or agreed that patients would contact therapists more with questions about their notes and that therapists would need to spend more time writing documentation. Similarly, eight in 10 somewhat agreed or agreed that therapists would be less candid in their notes with the knowledge patients could read them. Despite this, around six in 10 somewhat agreed or agreed patients would find significant errors in their notes. In addition, most trainee therapists believed patients would worry more if open notes were implemented. Almost all students (94.1%) somewhat agreed or agreed that education about open notes should be part of psychotherapy training. However, trainees in Switzerland also forecast some potential benefits of open notes: around eight in 10 believed open notes would benefit informed consent processes and for patient communication, and three quarters of those surveyed somewhat agreed or agreed that making open notes available to psychotherapy patients is a good idea. Similarly, around three in four participants somewhat agreed or agreed that patients would trust their therapist more and feel more in control over their own healthcare because of access.

Opinions on the effects on patients and therapists were further elucidated by the open comments. Four broad themes emerged: (a) negative impact on therapy; (b) positive impact on therapy; (c) impact on patients; and (d) documentation. Trainees identified concerns around increased workload, increased pressures on therapists, and harms to the therapy process and therapeutic relationship. They expressed concerns about patients’ feeling confused or anxious, of feeling misunderstood, judged, or offended. However, some trainees identified benefits with respect to the psychotherapy process, including identifying shared goals, strengthened patient autonomy, and increased transparency. Respondents also described a variety of challenges related to documentation including changes in note taking, and the need for “shadow records” after implementation whereby therapists would curate a separate, private record written specifically for themselves. Qualitative analysis was applied to explore perceptions of trainees on the impact of open notes on different types of therapy. A major theme was that the innovation would not be implemented the same way for distinctive modalities, with some trainees anticipating that the approach would be more difficult to implement for some therapies, such as psychoanalysis. In addition, participants reported that open notes would differ depending on patient capabilities and needs and could prove more challenging depending on the diagnosis or among patients with acute illnesses.

### Comparison with previous work

The findings of this study are consistent with that of other surveys undertaken in open notes in mental healthcare. Similar to other surveyed mental health clinicians,^19–21,29^ psychotherapy trainee were sometimes positive about open notes believing transparency and trust could be strengthened (“*In principle, it is a good thing but*…”^22,36^). However, like the only qualitative study into psychotherapy professionals’ views on the practice, attitudes were tempered by concerns about the risk of patient confusion and offense, and of compromising the psychotherapy relationship after access.^22^ In the Chimowitz study, participants were generally positive sharing access suggesting it could improve candor in clinical sessions. They reported few disruptions to their workload but admitted reluctance about discussing notes with patients during sessions. As the study authors noted, participants also tended to refer to open therapy notes mostly in hypothetical language believing most patients would not read what they wrote. Like the opinions of trainees in our study, their experiences suggested a lack of confidence about using the notes in psychotherapy processes. While the Chimowitz study explored the views of therapists who used psychodynamic and cognitive behavioral therapy approaches, it did not examine differential perspectives on these approaches to documentation. Our study is the first to identify potential challenges, including in documentation practices, of using open notes across distinct talking therapy modalities.

In our study, trainees identified the potential need for a shadow record to document their psychotherapy notes. This echoes findings among other mental health clinicians who report keeping a shadow record to ensure that they preserve the necessary detail in notes without risking offense or confusion. In a Norwegian study, healthcare professionals noted they kept shadow records on paper or their own computer.^32^ Beyond mental health contexts, although some clinician surveys have been undertaken,^29,37^ very little is understood about how ORA might objectively modify the nature of records including the detail, documenting differential diagnoses, or of the use of accessible and sensitive language in notes.^38^

Previous research in psychotherapy ethics has highlighted the problems associated with informed consent in psychotherapy,^39–43^ including the routine failure to provide pertinent and accessible information about psychotherapy processes and the value of specific treatment techniques. Recently, it has been proposed that open notes might provide a new tool in psychotherapy to help strengthen patient autonomy by providing a platform to extend opportunities to explain treatment techniques and processes.^44^ Despite identifying the potential for confusions associated with technical language or jargon in psychotherapy documentation, trainees in our study also anticipated open notes could be a useful vehicle for strengthening patient autonomy, transparency, and informed consent processes.

More than six in 10 trainees believed that a majority of patients might find significant errors in their psychotherapy notes.^45^ This prediction is consistent with other studies which suggest that mental health clinicians and general practitioners also believe that patients will identify errors and omissions in their notes.^19,37^ In a study by Bell et al. in the USA, of more than 22,000 out-patients who read their notes at three diverse health systems, around one in five reported finding an error with 40 percent perceiving the mistake as serious.^46^ Failure to correct errors, or to address potential omissions means both patients and therapists may be relying on inadequate tools,^1^ which may further exacerbate the potential for misinterpretations and clinical errors.^47^

Trainees in our study also suggested that it might not be appropriate to open notes to some psychotherapy patients, depending on their condition or other patient factors. Participants’ uncertainties about when it is appropriate to share access, including whether there are occasions when access might be unsuitable, echo concerns identified by other clinicians. In recent qualitative work exploring open notes in mental healthcare which surveyed patients, clinicians, and researchers, respondents agreed that failure to offer access to some patients, however, could exacerbate stigmatization creating its own harms, or lead to inappropriate decisions about whom to offer access.^19,20^ Currently, there is a lack of evidence-based policy, and lack of research which is focused exclusively on examining the experiences of sharing notes with patients with severe mental illnesses, addiction disorders, personality disorders, or those in hospitalized psychiatric care.^23,24^

### Strengths and limitations

This is the first survey of psychotherapy trainees’ familiarity with, and opinions about, the use of open notes in psychotherapy practice. A particular strength of the study is the diversity of psychotherapy modalities which participant trainees reported intending to practice.

This study has several limitations. Although the response rate was reasonable for an online survey (35.82%), the restricted sample size and restriction to one academic center limits generalizations about trainees’ views. The survey was administered during the COVID-19 pandemic, and it is unknown whether this affected response rates, or response biases among our respondents. Comments to free-text questions were brief—only 1 or 2 sentences or written in bullet points—restricting a more in-depth understanding of respondents’ opinions. The free-text responses were more negative than the structured items, a predictable pattern in surveys where the methods are combined.^48^ Because the survey was administered online, it was not possible to obtain a more in-depth exploration of participants’ views which focus groups or interviews might have provided. Our participants were drawn from various levels of psychotherapy training, and potentially disaggregating their responses would have revealed differences in opinion; however, owing to the small sample size this was not practicable.

The majority of our participants did not use open notes in the past. Given the increasing international spread of ORA exploring the awareness and opinions of tomorrow's psychotherapists is pertinent. Rapid expansion of telemedicine and digital interventions in psychotherapy suggests patients and therapists will increasingly expect asynchronous web-based tools to support treatment techniques.

### Implications and future directions

We recommend that much more concentrated efforts are needed to solicit the views and experiences of open notes among practicing psychotherapists and clinical psychologists, including those who use different kinds of talking therapies. Experiences and best practices should also be summarized and included in the curriculum for the benefit of trainees to ensure that they are prepared for writing notes.^49,50^ Previous research suggests that faculty's views on open notes might shape trainees’ opinions.^51^ In tandem, given that clinicians’ perceptions of patients’ experiences with ORA are often at odds with patient's actual experiences,^1^ much more work is needed to explore the views of patients with accessing their psychotherapy notes online, including the benefits and harms of reading their documentation. Finally, advances in generative artificial intelligence including chatbots powered by large language models (LLMs) such as ChatGPT are set to change the frontiers of clinical documentation^52,53^ including in mental health contexts.^54^ These chatbots have strengths in summarizing complex information and have impressive abilities to attune writing styles and tone for different readership making them highly relevant for therapists writing open notes. However, LLMs also carry limitations—they tend to make things up (“hallucinations”) and can embed biases and unwanted stereotyping.^53^ Therefore, we strongly suggest that future research examine how psychotherapy intersects with LLM-powered chatbots, including the opinions and experience of therapists in using these tools.

## Conclusions

This mixed-methods study provides exploratory insights into the views of 72 psychotherapy trainees in Switzerland regarding the impact of open notes on patient care and psychotherapy practice. In general, trainees expressed mixed views about open notes. They identified many potential benefits including patient communication, education, and informed consent processes. However, they also identified concerns related to the potential for access to increase workload, harm the psychotherapeutic relationship, compromise the quality of records, and increase risk of shadow records.

Sharing psychotherapy notes is not routine in countries which have begun opening ORA to patients, including Switzerland. However, even in countries where access to therapy notes is not mandatory, many social workers and therapists report opening access to patients. Still, given the nature of the treatment processes and techniques, in psychotherapy contexts, open notes may invite unique challenges with respect to documentation, including potential risks of harm or offense, balanced with respect for patient transparency and autonomy, and more focused work is now needed to understand the particular challenges in this domain. Psychotherapists working with different therapeutic approaches will also need advice and guidance, including formal training, to become more comfortable writing and talking about documentation that patients may read, including how to manage disagreements, perceived errors, and feedback.

## Supplemental Material

sj-docx-1-dhj-10.1177_20552076241242772 - Supplemental material for Open notes in psychotherapy: An exploratory mixed methods survey of psychotherapy students in Switzerland Supplemental material, sj-docx-1-dhj-10.1177_20552076241242772 for Open notes in psychotherapy: An exploratory mixed methods survey of psychotherapy students in Switzerland by Anna Kharko, Sarah Buergler, Annika Bärkås, Maria Hägglund, Jens Gaab, Asbjørn Johansen Fagerlund, Cosima Locher and Charlotte Blease in DIGITAL HEALTH

sj-docx-2-dhj-10.1177_20552076241242772 - Supplemental material for Open notes in psychotherapy: An exploratory mixed methods survey of psychotherapy students in Switzerland Supplemental material, sj-docx-2-dhj-10.1177_20552076241242772 for Open notes in psychotherapy: An exploratory mixed methods survey of psychotherapy students in Switzerland by Anna Kharko, Sarah Buergler, Annika Bärkås, Maria Hägglund, Jens Gaab, Asbjørn Johansen Fagerlund, Cosima Locher and Charlotte Blease in DIGITAL HEALTH

sj-doc-3-dhj-10.1177_20552076241242772 - Supplemental material for Open notes in psychotherapy: An exploratory mixed methods survey of psychotherapy students in Switzerland Supplemental material, sj-doc-3-dhj-10.1177_20552076241242772 for Open notes in psychotherapy: An exploratory mixed methods survey of psychotherapy students in Switzerland by Anna Kharko, Sarah Buergler, Annika Bärkås, Maria Hägglund, Jens Gaab, Asbjørn Johansen Fagerlund, Cosima Locher and Charlotte Blease in DIGITAL HEALTH
