# Supplementary material for: Open notes in psychotherapy: An exploratory mixed methods survey of psychotherapy students in Switzerland
Source: Digit Health. 2024 Mar 28;10:20552076241242772. doi: 10.1177/20552076241242772 (PMC10981219; doi:10.1177/20552076241242772)
Supplement: sj-docx-1-dhj-10.1177_20552076241242772 - Supplemental material for Open notes in psychotherapy: An exploratory mixed methods survey of psychotherapy students in Switzerland [file sj-docx-1-dhj-10.1177_20552076241242772.docx]

LimeSurvey - ‘OPEN NOTES’ AND PSYCHOTHERAPY: ... [https://sec.psycho.unibas.ch/kppt/index.php/admin/printables...](https://sec.psycho.unibas.ch/kppt/index.php/admin/printables...1)

‘OPEN NOTES’ AND PSYCHOTHERAPY: OPINIONS OF PSYCHOTHERAPY TRAINEES AND PRACTITIONERS AT A SWISS UNIVERSITY

Dear Student,

Researchers at the Division of Clinical Psychology and Psychotherapy, University of Basel invite you to take part in a survey. We are inviting you, as psychotherapy/clinical psychology trainees and practitioners to give your opinions about ‘open notes’ in psychotherapy. We will provide you with information about this concept, and do not expect you to have any prior knowledge of it. This research will help to inform clinical psychology educators and policy-makers.

The survey should take around 10 minutes to complete. We will not collect any identifying information from
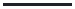
you, and your responses will be fully anonymous. Your response will be collated with those of other respondents in aggregated, anonymous form. ***This survey is not a test; we are interested in your*** ***opinions as clinical psychology and psychotherapy students.*** We refer you to the Information and Consent Sheet for more information.

If you decide to participate, we appreciate your time and contribution to our research. Thank you.

Dr Cosima Locher, University of Plymouth and University of Basel

Email contact: [cosima.locher@unibas.ch](mailto:cosima.locher@unibas.ch) (<mailto:cosima.locher@unibas.ch>) There are 29 questions in this survey

INFORMATION SHEET AND CONSENT FORM

[]

Thank you for considering participating in this research project. The purpose of this document is to explain to you what the work is about and what your participation would involve, so as to enable you to make an informed choice.

The purpose of this study is to investigate the opinions of clinical psychology and psychotherapy professionals/trainees about ‘open notes’. ‘Open notes’ refers to the practice of sharing patients’ online access
to their clinical notes via secure patient portals. Open notes in healthcare invites patients to log in to a secure online health portal to read all of their electronic health record including the very words written by health professionals. A growing number of countries worldwide

[1](https://sec.psycho.unibas.ch/kppt/index.php/admin/printables...1) von 14 05.10.20, 17:28

LimeSurvey - ‘OPEN NOTES’ AND PSYCHOTHERAPY: ... [https://sec.psycho.unibas.ch/kppt/index.php/admin/printables...](https://sec.psycho.unibas.ch/kppt/index.php/admin/printables...2)

allow patients to access these clinical notes. However, there is still debate about whether patients should be allowed access to their psychotherapy notes. In this survey we do not expect you to have any prior knowledge about open notes. Instead, we are

interested in your opinions about the potential effects of this practice in psychotherapy.

Should you choose to participate, you will be asked
some non-identifying demographic information,

followed by some short answers requesting your opinions. Some questions request your to select

answers from multiple options and some are open-
ended comment boxes asking you to express your opinions on how open notes might impact
psychotherapy. The survey also asks about your familiarity with open notes. The survey will take around 10 minutes to complete.

Your responses will help us to better understand the opinions of future clinical psychology and psychotherapy professionals/trainees on open notes, and may help to inform psychotherapy-training curricula. While there are no direct benefits to you, participating in this survey
may help to stimulate you to think about the role of
open notes in psychotherapy.

Participation in this study is completely voluntary. There is no obligation to participate, and should you choose to do so you can refuse to answer specific questions, or decide to withdraw from the study. We will not collect any personal or sensitive information therefore the survey will be fully anonymous. The survey is not a

test, and your decision to participate will not affect your grades. Ticking the box below will indicate consent to participate.

You maintain the right to withdraw from the study at
any stage up to the point of data submission. At this point your data will be collated with that of other participants and can no longer be retracted.

The anonymous data will be stored securely for up to
ten years on the University of Basel Server in secured form. The information you provide may contribute to research publications and/or conference presentations which may be publicly available. However, your contributions will be fully anonymous.

[2](https://sec.psycho.unibas.ch/kppt/index.php/admin/printables...2) von 14 05.10.20, 17:28

LimeSurvey - ‘OPEN NOTES’ AND PSYCHOTHERAPY: ... [https://sec.psycho.unibas.ch/kppt/index.php/admin/printables...](https://sec.psycho.unibas.ch/kppt/index.php/admin/printables...3)

We do not anticipate any negative outcomes from participating in this study.

This study has obtained ethical approval from the Research Ethics Committee at the University of Basel

If you have any queries ab[out this research, you can](mailto:cosima.locher@unibas.ch) [contact Dr Cosima Locher: cosima.locher@unibas.ch](mailto:cosima.locher@unibas.ch)
([mailto:cosima.locher@unibas.ch)](mailto:cosima.locher@unibas.ch)

If you agree to take part in this study, please complete the consent form overleaf.

**Do you consent to participate in this study?**

If you select ‘No’ you will be forwarded to the end of the survey.

*

Please choose **only one** of the following:


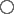
 Yes

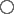
 No

[3](https://sec.psycho.unibas.ch/kppt/index.php/admin/printables...3) von 14 05.10.20, 17:28

LimeSurvey - ‘OPEN NOTES’ AND PSYCHOTHERAPY: ... [https://sec.psycho.unibas.ch/kppt/index.php/admin/printables...](https://sec.psycho.unibas.ch/kppt/index.php/admin/printables...4)

SECTION A: DEMOGRAPHIC QUESTIONS

[]

In this section we will ask demographic questions. []A1: Gender. Please select a box. *

Choose one of the following answers

Please choose **only one** of the following:


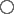
 Male


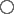
 Female


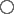
 Prefer not to answer


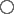
 Not listed above (please specify):

[]A2: Year of birth. Please choose the date. *

Please enter a date:

[]A3: Are you a student? *

Please choose **only one** of the following:


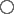
 Yes

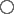
 No

[]A4: Please list your highest degree, and your current program of study. *

**Only answer this question if the following conditions are met:**
Answer was 'Yes' at question '5 [A3student]' (A3: Are you a student?)

Please write your answer(s) here:

Highest degree (e.g. Bachelors, Masters, PhD):

Program of study:

[]A5: Do you intend to practice as a psychotherapist? *

**Only answer this question if the following conditions are met:**
Answer was 'Yes' at question '5 [A3student]' (A3: Are you a student?)

Choose one of the following answers

Please choose **only one** of the following:


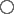
 Yes


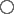
 No

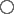
 Unsure

[]A6: Please list the primary modality you intend to practice (please select all that apply). *

[4](https://sec.psycho.unibas.ch/kppt/index.php/admin/printables...4) von 14 05.10.20, 17:28

LimeSurvey - ‘OPEN NOTES’ AND PSYCHOTHERAPY: ... [https://sec.psycho.unibas.ch/kppt/index.php/admin/printables...](https://sec.psycho.unibas.ch/kppt/index.php/admin/printables...5)

**Only answer this question if the following conditions are met:**

Answer was 'Yes' at question '5 [A3student]' (A3: Are you a student?) *and* Answer was 'Yes' at question '7 [A5psychotherapist]' (A5: Do you intend to practice as a psychotherapist?)

Check all that apply

Please choose **all** that apply:


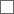
 Cognitive behavioral therapy

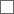
 Integrative therapy

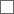
 Psychodynamic therapy

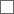
 Eclectic therapy

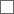
 Person-centered therapy

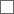
 Systemic therapy


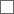
Other (not listed)::

[]A7: *FOR PRACTICING THERAPISTS:* Please list your

highest degree, and your current program of study. *

**Only answer this question if the following conditions are met:**
Answer was 'No' at question '5 [A3student]' (A3: Are you a student?)

Please write your answer(s) here:

Highest degree (e.g. Bachelors, Masters, PhD):

[]A8: *FOR PRACTICING THERAPISTS*: Please list the primary modality that you practice (please select all that apply). *

**Only answer this question if the following conditions are met:**
Answer was 'No' at question '5 [A3student]' (A3: Are you a student?)

Check all that apply

Please choose **all** that apply:


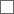
 Cognitive behavioral therapy

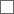
 Integrative therapy

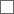
 Psychodynamic therapy

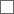
 Eclectic therapy

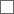
 Person-centered therapy

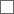
 Systemic therapy


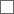
Other (not listed)::

[]A9: Please enter your nationality (optional).

Please write your answer here:

[]A10: What race/ethnicity do you primarily identify with. Please select (optional).

[5](https://sec.psycho.unibas.ch/kppt/index.php/admin/printables...5) von 14 05.10.20, 17:28

LimeSurvey - ‘OPEN NOTES’ AND PSYCHOTHERAPY: ... [https://sec.psycho.unibas.ch/kppt/index.php/admin/printables...](https://sec.psycho.unibas.ch/kppt/index.php/admin/printables...6)

Choose one of the following answers

Please choose **only one** of the following:


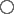
 Asian

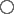
 Black

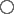
 White

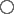
 Brown


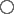
 Other

[]Thank you for completing Section A.

[6](https://sec.psycho.unibas.ch/kppt/index.php/admin/printables...6) von 14 05.10.20, 17:28

LimeSurvey - ‘OPEN NOTES’ AND PSYCHOTHERAPY: ... [https://sec.psycho.unibas.ch/kppt/index.php/admin/printables...](https://sec.psycho.unibas.ch/kppt/index.php/admin/printables...7)

SECTION B: OPEN NOTES AND PSYCHOTHERAPY PATIENTS

[]

The questions in this section are on your opinions about
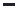
the potential impact of ‘open notes’ on psychotherapy
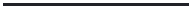
patients.

‘Open notes’ refers to the practice of sharing patients’ online access to their clinical notes via secure patient portals. Open notes in healthcare invites patients to log in to a secure online health portal to read their
electronic health record including the very words

written by health professionals. A growing number of countries worldwide allow patients to access these clinical notes. However, there is still debate about whether patients should be allowed access to their psychotherapy notes.

We do not expect you to have any prior knowledge
about open notes. Instead, we are interested in your opinions about the potential effects of this practice in psychotherapy.

[]B1: Please select a box. *

Please choose the appropriate response for each item:

disagree

1. In general, making open notes
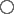
available to psychotherapy patients is a good idea.

somewhat disagree

somewhat
agree agree

| 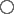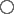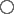2. Open notes will 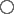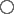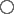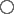increase efficiency in patient care in psychotherapy. |
| --- |
| 3. Open notes will improve patient 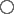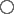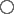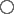satisfaction with their  psychotherapy care. |
| 4. Open notes will 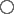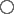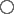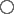enhance psychotherapy practice. |

[7](https://sec.psycho.unibas.ch/kppt/index.php/admin/printables...7) von 14 05.10.20, 17:28

LimeSurvey - ‘OPEN NOTES’ AND PSYCHOTHERAPY: ... [https://sec.psycho.unibas.ch/kppt/index.php/admin/printables...](https://sec.psycho.unibas.ch/kppt/index.php/admin/printables...8)

disagree

somewhat disagree

somewhat
agree agree

5. Open notes will


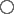

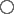

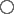

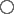
adversely impact

patient safety in

psychotherapy.

6. Open notes will

be useful for


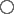

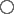

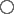

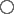
patient

communication in

psychotherapy.

7. Open notes will


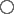

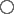

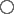

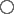
be useful for

patient education.

8. Open notes will

be useful for


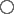

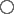

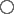

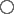
informed consent

processes in

psychotherapy.

[]B2: Among psychotherapy patients who read their therapists’ notes…

A majority of patients will… *

Please choose the appropriate response for each item:

disagree

…better

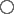
understand their mental health.

somewhat disagree

somewhat
agree agree

| 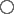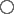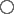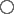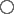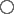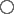…worry more. |
| --- |
| …feel more in 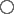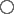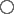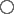control of their healthcare. |
| …disagree with 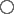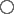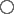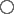what is written in their notes. |
| …request changes 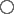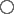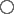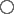to the content of their notes. |
| 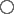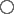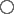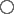…take better care of themselves. |
| …be more likely to 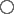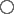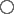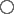make progress in therapy. |
| …be more likely to 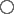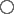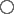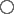adhere to treatment recommendations. |

[8](https://sec.psycho.unibas.ch/kppt/index.php/admin/printables...8) von 14 05.10.20, 17:28

LimeSurvey - ‘OPEN NOTES’ AND PSYCHOTHERAPY: ... [https://sec.psycho.unibas.ch/kppt/index.php/admin/printables...](https://sec.psycho.unibas.ch/kppt/index.php/admin/printables...9)

disagree

somewhat disagree

somewhat
agree agree

…be more likely to

take any mental


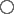

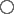

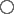
health-related

medications as

prescribed.

…find significant

errors in the notes.

…be better

prepared for

psychotherapy

sessions.

…trust their

therapist more.

…contact their

therapist more with

questions about

their notes.

…find the notes

more confusing

than helpful.

[]

B3: Please add any comments you may have on
the**potential harms or benefits** to patients of open notes in psychotherapy. *Please add one or two brief* *points. You can write in German or English.*

*

Please write your answer here:

[]Thank you for completing Section B.

[9](https://sec.psycho.unibas.ch/kppt/index.php/admin/printables...9) von 14 05.10.20, 17:28

LimeSurvey - ‘OPEN NOTES’ AND PSYCHOTHERAPY: ... [https://sec.psycho.unibas.ch/kppt/index.php/admin/printables...](https://sec.psycho.unibas.ch/kppt/index.php/admin/printables...10)

SECTION C: OPEN NOTES AND PSYCHOTHERAPISTS

[]

The questions in this section are on your opinions about the potential impact of ‘open notes’ on psychotherapists.

To recap: ‘Open notes’ refers to the practice of sharing patients’ online access to their clinical notes via secure patient portals. Open notes in healthcare invites
patients to log in to a secure online health portal to

read their electronic health record including the very words written by health professionals. A growing
number of countries worldwide allow patients to access these clinical notes. However, there is still debate about whether patients should be allowed access to their psychotherapy notes.

We do not expect you to have any prior knowledge
about open notes. Instead, we are interested in your opinions about the potential effects of this practice in psychotherapy.

[]

C1: If open notes were implemented in psychotherapy,
I expect that…

*

Please choose the appropriate response for each item:

disagree

…visits will take significantly longer.

somewhat disagree

somewhat
agree agree

| …psychotherapists will spend significantly more time addressing patient questions outside of visits. |
| --- |
| …patients who  read their notes will be offended. |
| …psychotherapists will be less candid  in their documentation. |

[10](https://sec.psycho.unibas.ch/kppt/index.php/admin/printables...10) von 14 05.10.20, 17:28

LimeSurvey - ‘OPEN NOTES’ AND PSYCHOTHERAPY: ... [https://sec.psycho.unibas.ch/kppt/index.php/admin/printables...](https://sec.psycho.unibas.ch/kppt/index.php/admin/printables...11)

disagree

somewhat disagree

somewhat
agree agree

…psychotherapists

will spend

significantly more

time writing and

editing their notes.

[]

C2: Please add any comments you may have on the **potential impact to psychotherapists**of open notes. *Please add one or two brief points. You can write in* *German or English.*

*

Please write your answer here:

[]

C3: ‘Open notes will be more challenging for some modalities of psychotherapy rather than others’. Please add any comments you may have on this statement. *Please add one or two brief points. You can write in* *German or English.*

*

Please write your answer here:

[]Thank you for completing Section C.

[11](https://sec.psycho.unibas.ch/kppt/index.php/admin/printables...11) von 14 05.10.20, 17:28

LimeSurvey - ‘OPEN NOTES’ AND PSYCHOTHERAPY: ... [https://sec.psycho.unibas.ch/kppt/index.php/admin/printables...](https://sec.psycho.unibas.ch/kppt/index.php/admin/printables...12)

SECTION D: OPEN NOTES: FAMILIARITY

[]

The questions in this section ask about your prior familiarity with open notes.

[]

D1: Prior to this survey, had you heard of the concept of ‘open notes’?

*

Please choose **only one** of the following:

Yes
 No

[]

D2: Have you ever accessed your own clinical notes online as a patient whether medical or mental health?

*

Please choose **only one** of the following:

Yes
 No

[]

D3: What is your opinion on the following statement?

*

Please choose the appropriate response for each item:

disagree

somewhat disagree

somewhat
agree agree

"Psychotherapy

training should

include education

about open notes."

[]

D4: Please add any other comments you may have about open notes or this survey.

Please write your answer here:

[12](https://sec.psycho.unibas.ch/kppt/index.php/admin/printables...12) von 14 05.10.20, 17:28

LimeSurvey - ‘OPEN NOTES’ AND PSYCHOTHERAPY: ... [https://sec.psycho.unibas.ch/kppt/index.php/admin/printables...](https://sec.psycho.unibas.ch/kppt/index.php/admin/printables...13)

[]Thank you for completing Section D.

[13](https://sec.psycho.unibas.ch/kppt/index.php/admin/printables...13) von 14 05.10.20, 17:28

LimeSurvey - ‘OPEN NOTES’ AND PSYCHOTHERAPY: ... [https://sec.psycho.unibas.ch/kppt/index.php/admin/printables...](https://sec.psycho.unibas.ch/kppt/index.php/admin/printables...14)

Thank you for taking the time to complete this survey.

Contact [cosima.locher@unibas.ch](mailto:cosima.locher@unibas.ch) (<mailto:cosima.locher@unibas.ch>) for any questions.

Submit your survey.

Thank you for completing this survey.

[14](https://sec.psycho.unibas.ch/kppt/index.php/admin/printables...14) von 14 05.10.20, 17:28
