# Supplementary material for: Open notes in psychotherapy: An exploratory mixed methods survey of psychotherapy students in Switzerland
Source: Digit Health. 2024 Mar 28;10:20552076241242772. doi: 10.1177/20552076241242772 (PMC10981219; doi:10.1177/20552076241242772)
Supplement: sj-docx-2-dhj-10.1177_20552076241242772 - Supplemental material for Open notes in psychotherapy: An exploratory mixed methods survey of psychotherapy students in Switzerland [file sj-docx-2-dhj-10.1177_20552076241242772.docx]

**Supplementary Material 2.**

**Reflexivity Statements**

AK, SB, and AB were directly involved in the qualitative analysis. AK is a female postdoctoral researcher working in Sweden. She has no prior professional or personal experiences with psychotherapy. AK has both professional and personal experience with open notes. As a researcher, AK has carried out several investigations of open notes in mental healthcare and patient ORA in general. As a patient in Sweden, AK has read the notes written about her care by healthcare professionals in primary and hospital care and considers the practice to be beneficial.

SB is SB is a doctoral student from Switzerland. She is a practicing psychotherapist in continuous training and has professional and personal experience in outpatient psychotherapy. As a researcher, SB has no experience with open notes. As a psychotherapist, however, she is familiar with the topic of open notes; however, SB has had no experience with open notes as a patient in Switzerland. SB regards the practice to be beneficial, but at the same time recognizes the associated challenges.

AB is a female doctoral candidate working in Sweden, focusing on evaluating and analyzing users' experiences of open notes in mental healthcare (i.e., "users" as patients and healthcare professionals). The context of mental healthcare in Sweden is holistic, including healthcare professionals such as physicians, psychologists, nurses, psychotherapists, physical and occupational therapists, social workers, and medical secretaries in outpatient care, inpatient care, and psychotherapy care. As a doctoral candidate, AB has carried out several studies on users' experiences with ORA in mental healthcare; however, AB has no professional or personal experience with psychotherapy. AB has, however, a general experience with open notes as a patient in Sweden.
